# Routine antenatal ultrasound by nurse-midwives in rural Kenya: a pragmatic trial assessing feasibility and effects of the *Mimba Yangu* (My Pregnancy) project

**DOI:** 10.3389/fgwh.2025.1555547

**Published:** 2025-09-09

**Authors:** Claudia Hanson, Lucy Nyaga, Nidhi Leekha, Michaela Mantel, Sarah Kedenge, Caroline W. Gitonga, Violet Naanyu, Jasmit Shah, Marleen Temmerman

**Affiliations:** 1 Centre of Excellence in Women and Child Health, Aga Khan University, Nairobi, Kenya; 2 Department of Global Public Health, Karolinska Institutet, Stockholm, Sweden; 3 Department of Imaging and Diagnostic Radiology, Aga Khan University, Nairobi, Kenya; 4 The Health Associates GmbH, Berlin, Germany; 5 Philips East Africa Limited, Nairobi, Kenya; 6 Philips Foundation, Nairobi, Kenya; 7 School of Arts and Social Sciences, Moi University, Eldoret, Kenya; 8 Brain and Mind Institute, Aga Khan University, Nairobi, Kenya; 9 Department of Internal Medicine, Aga Khan University, Nairobi, Kenya; 10 Department of Obstetrics and Gynecology, Ghent University, Ghent, Belgium

**Keywords:** ultrasound in early pregnancy, task-sharing, antenatal care, primary healthcare, positive pregnancy experience, maternal and newborn health

## Abstract

**Introduction:**

Task-sharing of obstetric ultrasound between nurse-midwives and doctors has the potential to operationalize the World Health Organization’s recommendation of at least one ultrasound before 24 weeks of gestational age for every pregnant woman. Here, we report on the feasibility, acceptability, and effects of the Mimba Yangu (My Pregnancy) task-sharing approach in rural Kenya.

**Methods:**

We conducted a pragmatic trial including 28 primary care facilities between April 2021 and March 2022, selected based on feasibility criteria. Fourteen facilities received the ultrasound intervention composed of (i) task-sharing with nurse-midwives, (ii) the use of portable ultrasound devices (Lumify™) connected to a tablet, and (iii) a digital platform facilitating distant support. Hybrid training of 32 nurse-midwives was provided based on a nationally derived curriculum, including theoretical and hands-on components, by an academic team. We used (i) in-depth interviews with nurse-midwives and healthcare managers, (ii) exit interviews using a quantitative questionnaire with pregnant and recently delivered women, and (iii) data abstraction from the health facility records. We descriptively analyzed data and used a difference-in-difference analysis based on a generalized linear model to assess the effect of the intervention on the number of antenatal visits.

**Results:**

The intervention was successfully and consistently implemented during a 9-month period in all 14 health facilities providing obstetric ultrasound services to 2,799 pregnant women. Interviews with trained nurse-midwives indicated that the intervention was relevant, feasible, and acceptable. In the intervention facilities, 50.4% of pregnant women received at least one ultrasound compared with 19.2% in the comparison facilities, where women were referred to other facilities for an ultrasound based on obstetric risk factors.

**Conclusion:**

Our analysis provides evidence of the feasibility, acceptance, and positive effects on service availability of providing ultrasound at the primary care level delivered by nurse-midwives. Scalability and feasibility of such an intervention are critical to global health but will demand policy reforms to allow task-sharing at national and sub-national levels.

## Introduction

Maternal and perinatal health continues to improve globally, but at a pace too slow to reach the United Nations Sustainable Development Goals (1). Improved antenatal care (ANC) has the potential to reduce the stagnant high global maternal mortality ratio and the high rates of stillbirth and neonatal deaths (2, 3). The latest World Health Organization ANC guidelines proposed an intensified scheme with eight contacts, including one ultrasound examination before 24 weeks of gestational age. The ultrasound examination is recommended to (i) measure gestational age; (ii) improve detection of fetal anomalies and multiple pregnancies; (iii) reduce induction of labor for post-term pregnancy; and (iv) improve a woman's pregnancy experience and maternal and newborn health outcomes (4).

In most high-income countries, at least one routine ultrasound examination during ANC is recommended (5). In low- and middle-income countries (LMIC), the scale-up of ultrasound has been challenging. Recent developments, however, offer new opportunities: electricity is now available in many primary facilities even in LMICs, and several portable low-cost ultrasound devices are on the market, allowing so-called point-of-care ultrasound (POCUS) examination, with the potential to integrate it into routine care (6). The key remaining challenges are (i) training of human resources to use ultrasound devices, (ii) supervision and quality assurance, and (iii) regulatory frameworks and policy guidelines. Highly trained staff, such as doctors or sonographers, are limited in number and typically work only in hospitals and urban or semi-urban areas (7). Instead, nurses and midwives are the main ANC providers at the primary healthcare level in LMIC (8), which is why, in our view, task-sharing of ultrasound imaging is the only scalable approach to make the services widely available and accessible. Task-sharing with nurses and sonographers is increasingly proposed not only for obstetric services but for a variety of clinical services (9). Furthermore, educating and training those who provide routine services at primary healthcare levels allows the integration of ultrasound imaging at the point of care, supporting the goal of providing people-centered integrated care (9). Point-of-care strategies providing remote support and supervision have been described as particularly useful to scale up ultrasound services (10).

Kenya is a lower middle-income country with continuously high estimated maternal and neonatal mortality rates (2, 3). The 2012 Kenyan National ANC guidelines recommend referral for fetal ultrasound assessment in cases of pregnancy complications, as limited human resources at ANC facilities make routine services challenging (11). According to the guidelines, obstetric ultrasound services in Kenya are provided at referral hospitals by trained medical doctors and/or professional radiologists/sonographers, while routine ultrasound during ANC is not strategized as yet.

Given the importance of advancing ultrasound in early pregnancy for improved outcomes, we undertook the *Mimba Yangu* project to test a scalable approach of providing ultrasound within routine ANC implemented by nurse-midwives in Kilifi County, Kenya. Here, we report on the feasibility, acceptability, and effects of the intervention on overall uptake of ANC and ultrasound use. Additional papers will report on the quality and accuracy of the ultrasound investigations.

## Method

We conducted a quasi-experimental four-arm trial involving 28 primary care facilities between April 2021 and March 2022 in Kilifi County, Kenya. The four arms consisted of 28 facilities and their catchment areas, which received either (i) ultrasound imaging (seven facilities), (ii) ultrasound imaging together with mobile obstetric monitoring (MOM) delivered at community level (seven facilities), (iii) MOM only (seven facilities), or (iv) standard healthcare (seven facilities). Intervention and comparison facilities were selected together with sub-national health authorities based on the criteria of feasibility and availability of electricity. The MOM intervention was designed to support ultrasound use during routine ANC and utilized a smartphone-based application delivered by community health volunteers operating within the vicinity of the project health facilities. The MOM facilitated pregnancy registration and referral of pregnant women to ANC including referral for ultrasound. Here, we report only the results of the ultrasound component compared with no ultrasound.

### Setting and population

The study was implemented in purposively selected rural sub-counties of Kilifi (Ganze, Kaloleni, and Rabai). Kilifi County is a large, high-disease burden county with a neonatal death rate of 24 per 1,000 live births despite a high uptake of antenatal care (77% of pregnant women complete four or more visits) and good uptake of facility-based childbirth (85%) (12). The population mainly relies on subsistence farming.

The Kenyan healthcare system has a pyramidal structure. Primary care facilities constitute levels 1–3. Level 1 community health units have community health promoters mainly involved in health promotion. Level 2 and level 3 health centers provide basic preventive and curative services, including ANC. These primary facilities are complemented by a level 4 sub-county (first-line referral) hospital (13). ANC services within primary healthcare comprise (i) history taking, (ii) physical examination and laboratory tests, (iii) health promotion, including advice on nutrition, (iv) planning the birth, (v) information regarding pregnancy, postpartum contraception, (vi) breastfeeding counseling, and (vii) provision of nutritional supplements and maternal vaccination.

### The *Mimba Yangu* intervention

**Figure 1:**
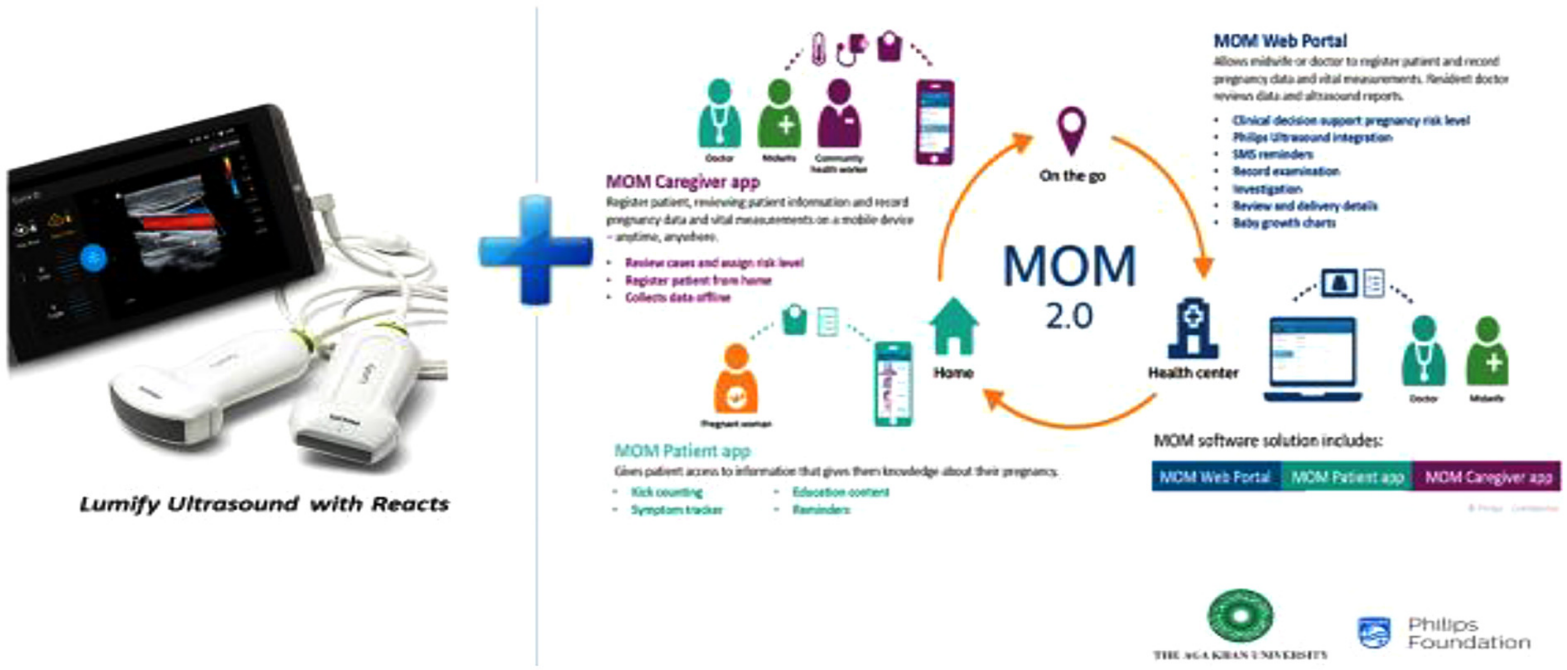
Digital intervention innovations of the Mimba Yangu project: Lumify™ portable ultrasound application. MOM, mobile obstetric monitoring; REACTS, remote education, augmented communication, training, and supervision.

The ultrasound intervention included two features: (i) a task-sharing approach where ultrasound was delivered by nurse-midwives using a portable ultrasound device (Lumify^™^) and (ii) a unique digital platform to facilitate distant support (6, 14) (Figure 1). This digital platform called *Remote Education, Augmented Communication, Training and Supervision* (REACTS) connected the ultrasound device to a tablet and facilitated Internet access and wider support. This support system included options for real-time image display, storage, and automatic transmission known as a *Picture Archiving and Communication System* (PACS). This system allowed professional verification, quality assurance of all ultrasound scans, and generation of ultrasound reports for the respective clients (15).

The ultrasound intervention was introduced through a three-week training program for nurse-midwives conducted during 11 September 2020 to 5 February 2021 at the participating facilities. The training was interrupted by the Kenyan nurses’ strike (December 2020 to February 2021) and the COVID-19 pandemic. The training was facilitated by licensed radiologists and obstetricians from the Aga Khan University (AKU) Hospital in Nairobi and one licensed obstetrician gynecologist from the Kenyan government and was supported by fourth-year Obstetrics and Gynecology residents employed at the AKU Hospital. The training was delivered partly remotely through an e-health platform established in a level 4 health facility. This hybrid training was not originally planned but necessary due to COVID-19 restrictions.

The curriculum, jointly designed by the AKU Nairobi and Philips, targeted nurse-midwives with no previous ultrasound training and covered (i) basic knowledge including the Lumify^™^ probe, (ii) an introduction to general ultrasound principles, (iii) ultrasound physics, and (iv) obstetric-specific ultrasound. It incorporated recommendations fom the International Society of Ultrasound in Obstetrics and Gynecology (ISUOG) Education Committee for basic obstetric and gynecological ultrasound training (16), along with learnings from previously implemented and tested modelsapproved by both the Nursing Council of Kenya and the Kenya Association of Radiologists (17, 18).

The theoretical training was followed by hands-on obstetric scanning in the sub-county hospital. Health workers completed written and practical examinations designed to assess both theoretical knowledge and practical skills. Staff tutored the nurse-midwives using the portable tablet, modem, and other practical issues related to transferring images and reports from the rural facilities to the PACS system.

Starting from April 2021 (after the nurses’ strike) ultrasound examinations were performed whenever a woman presented for her first ANC visit, preferably before 24 weeks of gestational age. Participants were informed about the ultrasound examination and assured that no immediate, delayed, or long-term risks of ultrasound imaging are known. Women received a copy of their scan report while the images were stored on the tablet and uploaded to the PACS system for validation using a detailed quality assurance checklist by study radiologists. Feedback was provided to the trained nurse-midwives through on-site supervision, hands-on training, and periodic virtual sessions.

The basic obstetric ultrasound screening technology was complemented by a unique REACTS digital platform developed and set up by Philips. The platform was designed to enable users to interact, teach, learn from, and assist each other. Additional on-site supervision, hands-on training, and mentorship of the nurses and midwives were conducted by the training facilitators and residents.

### Data collection and outcomes

**Qualitative interviews** to assess the feasibility and acceptability of the intervention, in-depth interviews with nurses, midwives, and health managers were conducted between 31 January and 31 February 2022. The interview guide was structured around the overall perceptions toward the intervention, experiences with providing ultrasound services to pregnant women during ANC, and the relevance and quality of the ultrasound training received. Interviews were conducted by trained social scientists. Recruitment and sampling followed a purposeful approach informed by information power and aiming at saturation (19). Thematic analysis was used to analyze the data (20).

**Quantitative data collection** to assess the effects of the intervention on the key outcomes of (i) uptake of ultrasound examination, (ii) timing and number of antenatal care visits, and (iii) satisfaction among women with services used two data sources: (1) exit interviews with pregnant women and those who delivered using a questionnaires with closed questions and (2) data abstraction from the health facility records and health management information system, i.e., Kenyan District Health Information System 2 (HMIS/DHIS-2) (21).

Exit interview surveys were performed from October 2020 to March 2021 (baseline) and January to March 2022 (endline) by trained data collectors supervised by the project data manager and the consultant biostatistician. The baseline interview period had to be extended to 5 months due to the Kenyan health workers’ strike (December 2020 and February 2021). Women attending ANC and or postnatal maternal and child healthcare (MCH) up to 6 months postpartum were invited to participate in the interviews. Data were collected through active recruitment during participants’ first visit to ANC or MCH clinics. Each day, 8–10 women aged 15–49 years attending either ANC or MCH clinics were approached by data collectors until the daily target sample size was reached. Data collection was conducted daily at level 3 and level 4 facilities. At some level 2 facilities with internal service schedules, data collection was performed only on days when ANC services were provided, where applicable. A structured questionnaire was administered including questions of ANC attendance, access to ultrasound services, and client satisfaction.

Data abstraction from ANC health facility records was conducted from October 2020 to December 2021 and again from January to March 2022. The data were abstracted from the primary paper-based ANC registers used at the facility level within the DHIS-2 and included (i) timing of ANC, (ii) total number of visits, and pregnancy characteristics such as parity and gravida.

### Allocation to intervention groups

The health facilities included in the intervention study were selected based on mutually agreed criteria instead of randomization due to logistical challenges, feasibility, and stakeholder requests. The selection criteria included government ownership, availability of maternal and child health services, availability of staff with at least two nurse-midwives, 24 h electricity, connectivity to 3G/4G network, and functional community health units. Comparison facilities were chosen based on similar criteria to the intervention arm facilities, but were located at a sufficient distance from the intervention areas to avoid contamination.

### Sample size

The sample size for the exit interviews was calculated based on the assumed outcome estimates of approximately 50%, such as the uptake of four ANC visits and satisfaction with care. The calculation assumed an effect size of 20%, *ρ* = 0.50%, and 80% power for two-sided hypothesis testing at a 0.05 significance level. The formula was used for non-randomized longitudinal difference-in-difference studies as proposed by Hu and Hoover (22). A minimum of 196 clients per intervention arm was proposed.

### Statistical analysis and data management

All collected data were entered into an electronic database using tablets and uploaded to the Open Data Kit (ODK) platform, which was managed by the project data manager. Summary statistics for the categorical variables were presented as frequencies and percentages. We used two comparison groups: (i) 14 facilities that received no ultrasound and (ii), as a *post hoc* sensitivity analysis, 7 facilities that received neither ultrasound nor MOM. The *post hoc* analysis comparing the 14 facilities receiving the ultrasound intervention with the 7 facilities with neither ultrasound nor MOM was conducted because community volunteers implementing the MOM had advised women to seek ultrasound services. Group comparisons between baseline and endline for the ultrasound and comparison group were computed using Fisher’s exact test. The difference-in-difference analysis was performed using a generalized linear model. This method was chosen to adjust for baseline differences as the clusters were not randomized. A *p*-value of <0.05 was considered statistically significant. Analysis was conducted using SPSS (IBM SPSS Statistics Version 20) and R (Version 4.3.1).

### Ethical considerations

The study protocol received ethical approval from the Aga Khan University, Nairobi Institutional Ethics Review Committee (IERC) and the National Commission for Science, Technology and Innovation (NACOSTI), Kenya. Kilifi County also approved and supported the study. Women included in the exit interviews were informed about the study and provided written informed consent. Minors between 15 and 18 years of age provided assent, while parents/guardians gave consent.

### Public involvement

The study was conceptualized, conducted, and disseminated with strong stakeholder participation.

## Results

**Figure 2:**
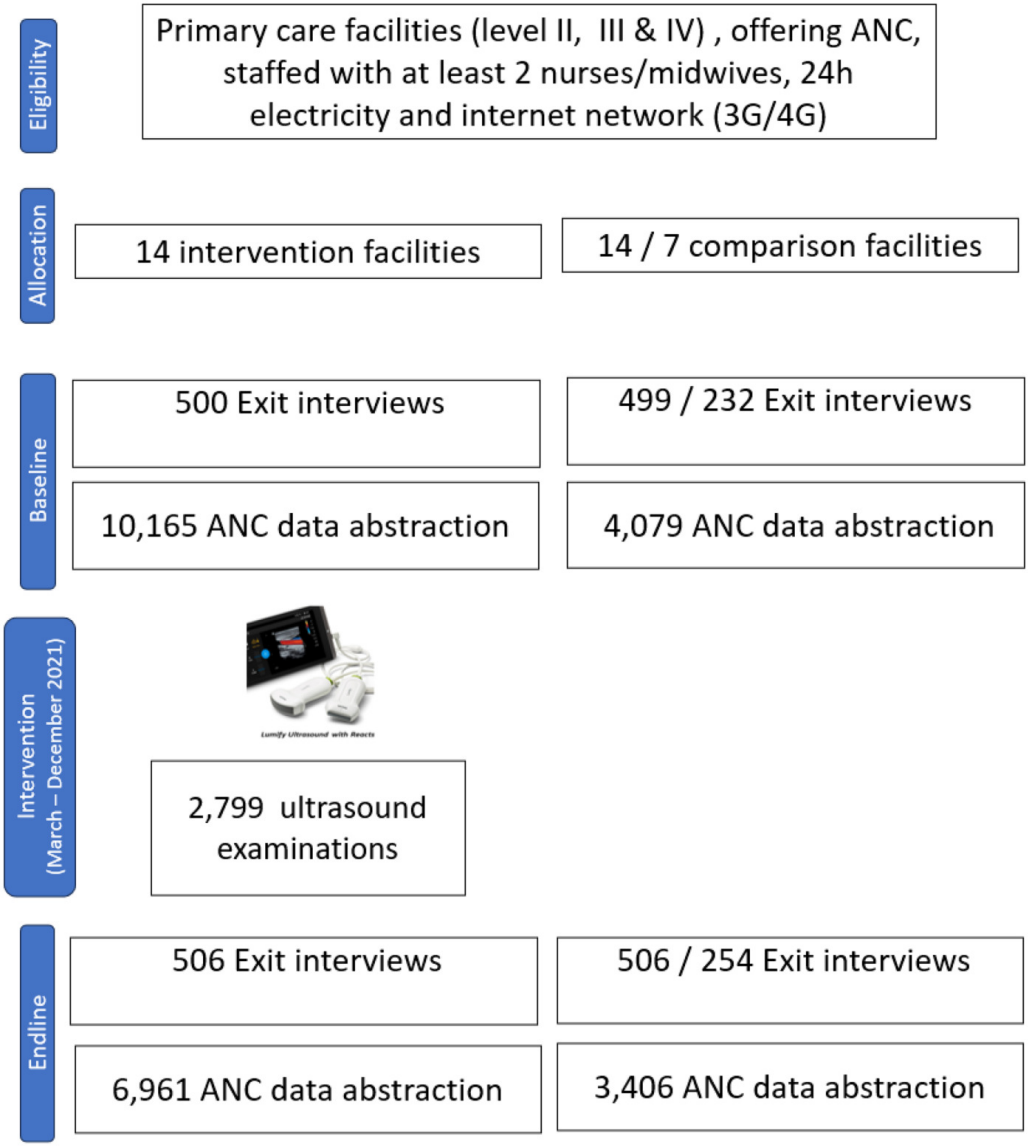
Trial flowchart.

The Mimba Yangu intervention was implemented in 14 intervention facilities (Figure 2). The comparison group of 14 facilities received standard care, where referral for ultrasound examinations to higher-level facilities in case of complications was promoted. A total of 2,799 pregnant women received an ultrasound examination between April and December 2021. At baseline, exit interviews were conducted with 500 women in intervention facilities and 499 in comparison facilities with no ultrasound. The *post hoc* analysis, which included only those facilities with neither ultrasound nor MOM, included 221 exit interviews. For the endline 506 exit interviews were conducted in each intervention and comparison facilities, 254 in comparison facilities with MOM.

A total of 31 nurse-midwives and health managers engaged in the qualitative interviews. Among them, 27 nurse-midwives had between 2 and 27 years of professional experience (mean age, 36.6 years), of whom 24 were diploma holders and 3 were degree holders. The four interviewed health managers were all diploma holders, with a mean age of 57 years and between 13 and 29 years of professional experience.

Providers and healthcare managers appreciated the intervention and clearly pointed out to the benefits of bringing ultrasound closer to the population:

Technology and innovation has helped mothers get these services at a lower level, because those scans were only done by the sub-county and the gynecologist…We used to think nurses cannot do ultrasound because we are considered to be in the village. The machine itself is portable [and serves] like the big one in the big facility. So this is so good, mothers are getting services in the village. (IDI, HCP, Kaloleni01)

Offering ultrasound was seen as a *pull* factor to increase the uptake of ANC in general:

Women were excited to see their unborn babies (1) and HCPs could identify any issues with pregnancy early enough (11) “… I've seen mother starting their ANC early, because they're excited. You see, sometimes you feel like, ‘What am I going to do? I'm a mother of three. I attended the ANC, I know the process, why should I go early?’ But now there is something new that is incorporated. So mothers now start their ANC early. It has improved our four ANC visits, because if this mother starts early, she will be finishing the fourth ANC. (IDI, Sub-county Manager, Kaloleni30)

However, the providers also raised challenging aspects, such as the inconsistent availability of ultrasound gel and paper or gauze for cleaning:

We get the reporting pads, enough gel and we have been given gloves and sanitizer. But the dignity pack* we spread it on the woman so that she does not get that gel. Because when you are doing a scan then the gel spreads. When you have it, and cover her, her clothes won't get dirty. It was there in the training. When we came to the ground, it was brought once. Just the two rolls till now. When you ask for it, they say that it is finished, and they are not budgeting on it. We make an effort, at times we use the serviettes, and we spread them, so that we can do the scan. At times if things are bad, you are forced to use a gauze roll and it is expensive… You have to improvise to do the scan. (IDI, HCP, Ganze18)

*The dignity pack consisted of Medi spread rolls and hand paper towels.

The uptake ultrasound provision, ANC, and satisfaction with services are displayed in Table 1.

**Table 1:** Uptake of antenatal care, ultrasound, and satisfaction at baseline and endline of 28 facilities included in the Mimba Yangu trial [source: exit interviews, October 2020 to March 2021 (baseline) and January to March 2022 (endline)].

Indicator	Category	Baseline				*p-*value	Endline				*p-*value	DID (95% CI)	*p-*value
		(*n* = 999)					(*n* = 1,012)						
		Comparison		Ultrasound			Comparison		Ultrasound				
Received ultrasound during this pregnancy	Yes	80	16.1%	96	19.2%	0.213	122	24.1%	254	50.4%	<0.001	23.2% (21.9%, 24.5%)	<0.001
	No	417	83.9%	404	80.8%		384	75.9%	250	49.6%			
Facility where ultrasound received	Current facility	10	12.5%	49	51.0%	<0.001	56	45.9%	206	81.1%	<0.001	−3.3% (−10.1%, 3.4%)	0.429
	Government dispensary	0	0.0%	3	3.1%	0.252	10	8.2%	5	2.0%	0.008	−9.4% (−16.0%, −2.7%)	1.000
	Government health center	1	1.3%	4	4.2%	0.378	17	13.9%	5	2.0%	<0.001	−14.9% (−20.9%, −8.9%)	0.008
	Government hospital	57	71.3%	21	21.9%	<0.001	28	23.0%	27	10.6%	0.003	37.1% (35.0%, 39.1%)	0.006
	Private clinic	3	3.8%	6	6.3%	0.513	4	3.3%	7	2.8%	0.753	−3.0% (−4.8%, −1.2%)	0.458
	Private hospital	9	11.3%	13	13.5%	0.819	7	5.7%	4	1.6%	0.044	−6.5% (−9.0%, −3.9%)	0.05
A healthcare professional making ultrasound	Doctor	74	92.5%	88	91.7%	0.582	40	32.8%	36	14.2%	<0.001	−17.8% (−21.6%, −14.0%)	0.119
	Nurse/midwife	4	5.0%	3	3.1%		79	64.8%	215	84.6%		21.8% (19.0%, 24.5%)	0.053
	Other/don't know	2	2.5%	5	5.2%		3	2.5%	3	1.2%		−4.0% (−6.4%, −1.5%)	0.203
Reason for ultrasound	Routine check	22	27.5%	26	27.1%	1.000	65	56.0%	124	49.6%	0.263	−6.0% (−8.0%, −4.1%)	0.697
	Nurse/ midwife advice	16	20.0%	27	28.1%	0.224	5	4.3%	57	22.8%	<0.001	10.4% (9.1%, 11.6%)	0.015
	Complication in pregnancy	33	41.3%	35	36.5%	0.537	42	36.2%	36	14.4%	<0.001	−17.0% (−20.2%, −13.8%)	0.019
	My wish	9	11.3%	6	6.3%	0.284	3	2.6%	31	12.4%	0.002	14.8% (11.5%, 18.1%)	0.004
	My husband’s/partner’s wish	0	0.0%	2	2.1%	0.501	1	0.9%	2	0.8%	1.000	−2.1% (−5.6%, 1.3%)	1.000
At least one ANC visit	Yes	496	99.4%	494	98.8%	0.506	504	99.6%	503	99.4%	1.000	0.4% (0.2%, 0.6%)	0.802
	No	3	0.6%	6	1.2%		2	0.4%	3	0.6%			
Number of ANC visits	4+	217	43.6%	183	37.0%	0.038	227	45.1%	246	49.0%	0.230	10.5% (9.9%, 11.1%)	0.175
	<4	281	56.4%	312	63.0%		276	54.9%	256	51.0%			
Satisfaction with services	Satisfied	489	98.6%	478	96.6%	0.061	498	98.8%	486	96.2%	0.016	−0.5% (−0.7%, −0.4%)	0.684
	Moderately satisfied	5	1.0%	15	3.0%		4	0.8%	16	3.2%			
	Dissatisfied	2	0.4%	2	0.4%		2	0.4%	3	0.6%			
Satisfaction with ultrasound	Satisfied	80	100.0%	92	95.8%	0.251	119	97.5%	249	98.0%	0.226	4.7% (−0.5%, −9.8%)	0.684
	Moderately satisfied	0	0.0%	2	2.1%		0	0.0%	3	1.2%			
	Dissatisfied	0	0.0%	2	2.1%		3	2.5%	2	0.8%			
Overall satisfaction with current services offered	Satisfied	498	99.8%	482	96.4%	<0.001	495	97.8%	493	97.4%	0.837	3.0% (1.8%, 4.2%)	0.013
	Mod satisfied	1	0.2%	16	3.2%		11	2.2%	13	2.6%			
	Dissatisfied	0	0.0%	2	0.4%		0	0.0%	0	0.0%			
Will recommend the current health facility	Yes	497	99.8%	489	98.0%	0.011	498	98.6%	502	99.2%	0.385	2.4% (1.3%, 3.5%)	0.019
	No	1	0.2%	10	2.0%		7	1.4%	4	0.8%			

The proportion of women receiving ultrasound imaging during ANC increased from 19.2% to 50.4% in intervention facilities and from 16.1% to 24.1% in comparison facilities [difference-in-difference 23.2 (95% CI: 21.9–24.5) percentage points, *p* < 0.001]. At baseline, ultrasound imaging was almost exclusively performed by doctors (92.5% in comparison facilities and 91.7% in intervention facilities), while at endline, 64.8% and 84.6% of ultrasound imaging in comparison and intervention facilities, respectively, was performed by nurse-midwives [difference-in-difference 21.8 (95% CI: 19.0–24.5) percentage points, *p* = 0.053].

The *post hoc* analysis performed to exclude facilities where the MOM intervention may have stimulated routine ultrasound through the community activities indicated a similar picture (Annex Table 1).

**Figure 3:**
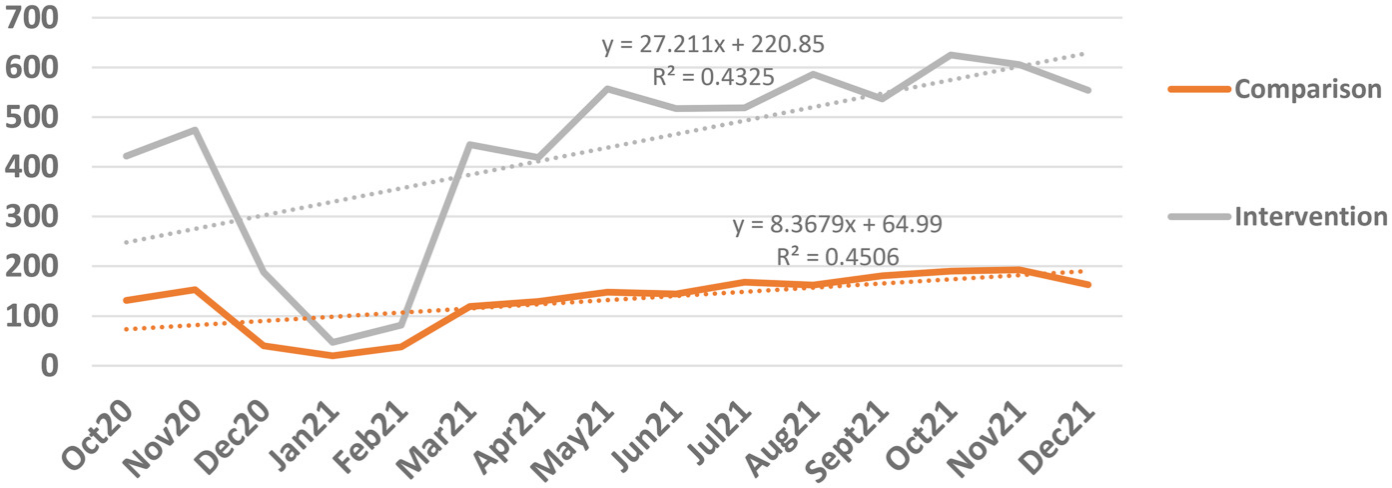
Temporal change of four or more ANC visits in the intervention and comparison (data abstraction of ANC services) facilities (the Kenyan nursing strike took place from December 2020 to February 2021) (source: data abstraction from health facilities, October 2020 to December 2021).

The ANC abstraction information (Figure 3) provided some evidence of an increased slope (increase by 0.272 compared with 0.084), thus a stronger increase in uptake of at least four ANC visits in the *Mimba Yangu* intervention compared with the comparison facilities.

## Discussion

This study evaluated routine basic obstetric ultrasound through task-sharing with nurse-midwives and found that the intervention brought ultrasound services closer to the community. In intervention facilities, 50.4% of women received at least one ultrasound imaging—with 84.6% of these performed by nurse-midwives—compared with 3.1% at baseline. Furthermore, our analysis suggested an increase in the utilization of four or more ANC visits in the intervention facilities compared with the comparison facilities.

The major increase in coverage with ultrasound services in the intervention facilities and the high number of investigations conducted support the feasibility and acceptability of the approach. Moreover, our qualitative interviews indicated that the nurse-midwives appreciated providing the ultrasound imaging. Similarly, in other studies, midwives expressed motivation and confidence in acquiring the knowledge, expressed their desire to perform ultrasound, and highlighted the empowerment created through making decisions without necessarily waiting for the availability of the doctor (23–25). Studies also attributed appreciation of skills to a prominent need and desire to gain further knowledge of the field (26, 27). Midwives in a study conducted in Ethiopia reported that the visual image of the baby produced by ultrasound facilitated communication with pregnant women (28). Nurse-midwives value the potential of ultrasound in decision-making (29).

Our results, suggesting higher coverage of at least four ANC visits during pregnancy in response to the ultrasound imaging, have also been proposed by other studies. An observational study evaluating point-of-care ultrasound in Ethiopia indicated an increased utilization of first and fourth ANC visits in primary facilities, comparing pre- and post-introduction periods using routine service statistics (30). Similarly, two studies in Uganda, a cluster-randomized trial advertising ultrasound and a pre-post assessment with non-randomized comparison facilities indicated an increase in ANC attendance (31, 32). However, a well-designed cluster-randomized trial in five LMIC countries, including Kenya, did not indicate increased use of ANC (33).

Tasking nurses and midwives with obstetric ultrasound is not a new idea, and several pilot studies have been implemented across LMIC (9, 34). No standard training curriculum has been established, and no preferred duration is recommended. In this study, which took place during the COVID-19 pandemic, the relatively short training period, the use of videoconferencing established at a primary referral hospital (level 4), and the distant support and hands-on supervision made this approach feasible and implementable. This project differed significantly from most other pilot projects, as a recent systematic review reported. Training sessions were typically delivered on-site, and the duration varied between very short trainings of only 3 days to 8 weeks (9). In Malawi, a short, 10-day training course was effectively implemented in six sites, and midwives reported feeling empowered by the new knowledge; however, insufficient continuous supervision was identified as a barrier to its effectiveness (27, 35). In our study, continuous supervision and hands-on training of the nurse-midwives were conducted on a regular rotating basis by our trainers, particularly the involved residents in Obstetrics and Gynecology, but the COVID-19 pandemic limited the total physical contacts.

In LMIC, routine obstetric ultrasound imaging at the primary healthcare level is not a standard operating procedure. Often, ultrasound during pregnancy is only available in urban settings, limited to private facilities, and delivered by radiologists. An approach such as our *Mimba Yangu* would demand policy reforms, underpinned by additional resources including (i) task-sharing of ultrasound imaging to nurses and midwifes, (ii) adoption of ultrasound training and curricula in the basic nursing and midwifery education programs, (iii) adoption of appropriate quality assurance processes including standard operation procedures/protocols at health facility levels, and (iv) technical support to ensure Internet availability and standardized safe data transmission procedures. An analysis of the costs and of the *Mimba Yangu* approach is outside of this paper. However, even if these changes were implemented, the present human resource crisis in LMIC would limit service availability (36).

### Strengths and limitations

A particular strength of our study was that the intervention covered a relatively large number of facilities compared with several other studies. The study area was a very typical rural place, which increases the relevance and applicability of the results to other LMIC settings. The introduction led to a consistent use of ultrasound, and 2,799 ultrasound examinations were performed in the 9 months of implementation of the initiative, indicating the scale of the pilot intervention. The evaluation used three methods of data collection and thus provided some triangulation of results. However, our analysis also has limitations. First, this is a quasi-experimental study where intervention and comparison facilities were not randomly assigned but chosen based on feasibility. This introduced a selection bias. We only assessed the approach on the provision of ultrasound examinations, ANC attendance, and patient satisfaction. Other trials have assessed the effect on referral, mortality, and morbidity and reported mixed results (9, 32, 33, 37). Furthermore, our qualitative interviews only targeted nurse-midwives, but not the doctors providing the support, which would have given an additional perspective on feasibility.

We have to assume contamination, and this may have weakened the differences between the intervention and comparison sites. The study areas were relatively closely situated to each other. The fact that women interviewed at the comparison facilities received an ultrasound by midwives hints that services may be sought at one of the ultrasound facilities once in pregnancy, while continuing ANC and child health services at the comparison facility. Our *post hoc* analysis, which excluded those facilities where community volunteers involved in the MOM intervention may have encouraged ultrasound services, indicated a similar picture pointing to some cross-contamination. The clear numerical trend to more uptake of ultrasound for routine examination or advice from a nurse/midwife indicates a very positive finding.

This study was conducted during the COVID-19 pandemic, which presented both challenges and special opportunities for the implementation and evaluation. In addition, data abstraction from ANC records took place when a nursing strike resulted in the closure of many facilities.

## Conclusions

Our study indicated the feasibility of routine basic ultrasound imaging in early pregnancy in rural facilities in Kenya through task-sharing of obstetric ultrasound services to the primary healthcare level. The relatively short training and the use of virtual support contributed to making this approach relatively feasible and practical to implement. We report that nurse-midwives are willing, motivated, and capable of providing these services under close supervision and mentorship. Our study confirmed that pregnant women are keen to access routine obstetric ultrasound screening during ANC visits. Thus, the scalability of this approach becomes a realistic option for local healthcare systems to bring routine basic ultrasound closer to the target population, potentially improving positive pregnancy experiences and maternal and newborn pregnancy outcomes as recommended by WHO. Our study adds to the increasing number of studies on the relevance of task-sharing as a feasible and potentially economic approach and supports policy changes and programming intentions.

## Data Availability

The raw data supporting the conclusions of this article will be made available by the authors upon reasonable request.
